# Microbial Community Structure and Methane Cycling Potential along a Thermokarst Pond-Peatland Continuum

**DOI:** 10.3390/microorganisms7110486

**Published:** 2019-10-24

**Authors:** Adrien Vigneron, Perrine Cruaud, Najat Bhiry, Connie Lovejoy, Warwick F. Vincent

**Affiliations:** 1 Centre d’études Nordiques (CEN), Université Laval, Québec, QC G1V 0A6, Canada; Najat.Bhiry@cen.ulaval.ca (N.B.); connie.lovejoy@bio.ulaval.ca (C.L.); warwick.vincent@bio.ulaval.ca (W.F.V.); 2 Département de Biologie, Université Laval, Québec, QC G1V 0A6, Canada; 3 Institut de Biologie Intégrative et des Systèmes, Université Laval, Québec, QC G1V 0A6, Canada; perrine.cruaud@gmail.com; 4 Takuvik Joint International Laboratory, Université Laval, Québec, QC G1V 0A6, Canada; 5 Département de Biochimie, de Microbiologie et de Bio-informatique, Université Laval, Québec, QC G1V 0A6, Canada; 6 Département de Géographie, Université Laval, Québec, QC G1V 0A6, Canada

**Keywords:** Archaea, Arctic, lakes, methane, methanogens, methanotrophs, permafrost, wetlands

## Abstract

The thawing of ice-rich permafrost soils in northern peatlands leads to the formation of thermokarst ponds, surrounded by organic-rich soils. These aquatic ecosystems are sites of intense microbial activity, and CO_2_ and CH_4_ emissions. Many of the pond systems in northern landscapes and their surrounding peatlands are hydrologically contiguous, but little is known about the microbial connectivity of concentric habitats around the thermokarst ponds, or the effects of peat accumulation and infilling on the microbial communities. Here we investigated microbial community structure and abundance in a thermokarst pond-peatland system in subarctic Canada. Several lineages were ubiquitous, supporting a prokaryotic continuum from the thermokarst pond to surrounding peatlands. However, the microbial community structure shifted from typical aerobic freshwater microorganisms (*Betaproteobacteria* and *Alphaproteobacteria*) in the pond towards acidophilic and anaerobic lineages (*Acidobacteria* and *Choroflexi*) in the connected peatland waters, likely selected by the acidification of the water by *Sphagnum* mosses. Marked changes in abundance and community composition of methane cycling microorganisms were detected along the thermokarst pond-peatland transects, suggesting fine tuning of C-1 carbon cycling within a highly connected system, and warranting the need for higher spatial resolution across the thermokarst landscape to accurately predict net greenhouse gas emissions from northern peatlands.

## 1. Introduction

Climate warming has led to the rapid degradation of permafrost soil across the subarctic and Arctic, inducing changes in area and distribution of vegetation, peatlands, lakes, and ponds [1,2]. Abrupt thawing of ice-rich permafrost, occurring in many parts of the Arctic landscape, causes surface collapse and produces basins [3] that then fill with meltwater, snow, and rainfall to form thermokarst lakes and ponds [4,5]. The latent heat of the water in these basins further thaws the underlying ground ice, leading to ongoing subsidence, deepening, and expansion of open water [6,7]. 

In permafrost peatlands, these waters contain high concentrations of colored dissolved organic matter derived from the thawed peat [8]. The lakes are, therefore, highly colored and absorb solar radiation during their open water period in summer, leading to thermal stratification and anoxic bottom waters [9]. These anoxic conditions favor anaerobic microbial taxa and metabolism, such as methanogenic organic matter degradation [10]. Thermokarst lakes are therefore productive bioreactors and an increasing source of greenhouse gas emissions [7,9,11,12]. With accelerated climate warming in the Arctic, the number and size of these lakes are increasing in certain areas, such as eastern Canada, whereas in other regions evaporation, drainage, sediment inputs, plant colonization, and peat accumulation are causing the disappearance of older lakes [2,5,13]. 

Thermokarst ponds are often characterized by high rates of peat accumulation [14,15], however water chemistry, lake morphometry, and moss species composition influence the rates of peatland formation [16]. Specifically, anaerobic conditions in the water, acidification by aquatic mosses and cold temperatures reduce the rate of organic matter decomposition, which results in primary production exceeding organic matter decomposition and leads to the substantial accumulation and sedimentation of incompletely decomposed organic matter or peat [16,17]. A by-product of this process is the generation of substrates for anaerobic bacteria and methanogens, with metabolism leading to release of methane to the atmosphere [18,19,20]. For example, in Stordalen mire (Sweden), peatland formation following permafrost thaw was accompanied by a change of organic carbon composition and enhanced rates of methane emission [21].

In both thermokarst lakes and peatlands, the produced methane can be oxidized by methanotrophic microbial communities before reaching the atmosphere, reducing the net greenhouse gas emissions. However, estimates of the fraction of methane that is consumed by this process is extremely variable, from zero to 100%, depending on local conditions [22,23,24,25]. Aerobic methane oxidation has been identified as the predominant methane consumption pathway in these environments [23], with known anaerobic methanotrophs being conspicuously undetectable in thermokarst lakes and peatlands [26]. High populations of methane oxidizers usually develop where methane and oxygen profiles overlap. In the shallow ponds of northern Quebec, aerobic methanotroph populations are also detected above the oxycline and into the anoxic layer of water columns, suggesting not only tolerance to anoxic conditions by some methanotrophs, but also a degree of gas exchange within the ponds [27]. However, the overall factors selecting for major groups and species of methanotrophs within thermokarst lake communities remain poorly understood. The marked differences in methanotroph community composition between organic and mineral type thermokarst lakes suggests that the source and characteristics of organic matter likely play a role [27]. In peatlands, the presence of *Sphagnum* mosses seems to influence the species composition and activity of methanotrophs [28], with specific acidophilic methanotrophs colonizing the dead, water-filled cells of the *Sphagnum*. A potential facultative syntrophic relationship between methanotrophs and *Sphagnum* has been proposed, whereby *Sphagnum* provides oxygen and methane, under pH conditions that are optimal for the bacteria. Conversely, methanotrophs provide methane-derived CO_2_ for photosynthesis, leading to an effective recycling of the methane [28,29].

The Sasapimakwananisikw River valley (SAS) is situated in the sporadic permafrost area of subarctic eastern Canada and is considered to be representative of the extensive permafrost peatlands that occur along the coast of eastern Hudson Bay (Canada). The region, initially colonized by peat-forming mosses and other plants, became frozen during the Little Ice Age to form an organic-rich permafrost [14,30]. Over the last 60 years, accelerated thawing of the permafrost has changed this subarctic landscape, and numerous thermokarst lakes and ponds have developed over the peatlands [2]. Previous investigations of thermokarst lakes in the region have shown strong greenhouse gas emissions [9,12] and the presence of both methanogenic and methanotrophic communities throughout the water column [31,32]. However, methane cycling microbes in the surrounding semi-aquatic peatland area and the connectivity (potential exchanges) between these two habitat types have not been explored to date.

The aim of this study was to evaluate gradients in microbial community composition and abundance in a permafrost wetland continuum, from the waters of a thermokarst pond to the waters underlying the surrounding peatlands. We aimed to test if the progressive colonization of thermokarst ponds by aquatic vegetation influences the microbial communities and, more specifically, the methane cycling potential (occurrence and abundance of methanogens and methanotrophs) that could affect the local carbon balance.

## 2. Materials and Methods 

### 2.1. Site Description, Sample Collection and Nucleic Acid Extraction

**Figure 1:**
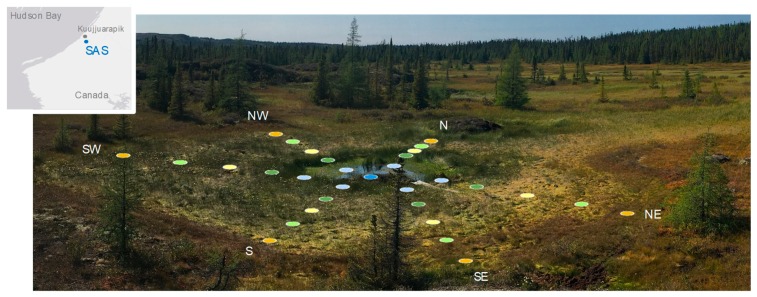
Photograph of thermokarst pond SAS2E in the subarctic peatland valley and the position of the sampling points. The distance between points on each transect is 2 m. The map insert shows the location of the valley (SAS) near the eastern coast of Hudson Bay, Quebec, Canada.

The SAS Valley study site lies in the sporadic permafrost zone of subarctic Québec, around 10 km southwest of Whapmagoostui-Kuujjuarapik, Canada (55°13.160′ N, 77°41.806′ W, Figure 1). It is part of a permafrost peatland characterized by palsas (1–7 m high mounds of peat rising out of the mire and containing a permafrost core of ice-rich peat or silt), thermokarst ponds and peatlands, covering approximately 5 km^2^ at a mean altitude of 110 m above sea level [14]. The thermokarst ponds are oval or circular shaped thaw lakes, from 5 to 20 m across. 

This study focused on thermokarst pond SAS2E and its surrounding peatland (Figure 1). This pond (referred to as B1 in [33]) is 8 m in diameter and 0.8 m deep, with a small collapsed palsa near its centre. In previous measurements of its surface waters in summer, it had a pH of 4.86, a high organic carbon content of 28 mg DOC L^−1^, total nitrogen of 1.4 mg L^−1^, a low dissolved oxygen concentration of 5.3 mg L^−1^ and a methane concentration at the surface of 2.3 µM, well above air-equilibrium and indicative of continuous methane emission [33]. The pond was centred on a 25-m diameter bog colonized by *Carex* sedges and *Sphagnum* mosses. The presence of permafrost mounds (palsas) around the study site resulted in a micro-topographic gradient that implied a decreasing water table and increasing acidity with distance from the centre [34]. This micro-topographic gradient was visible from the vegetation pattern and soil stability (Figure 1).

Six transects of five sampling points each from the thermokarst pond to its surrounding peatland area were carried out on 5 September 2017. The first point of the transect was sampled in the littoral water, 4 m from the centre of the pond, then in the peatland at 6, 8, 10, and 12 m away from the centre of the pond on north, south, northeast, southeast, northwest, and southwest axes (N, S, NE, SE, NW and SW, respectively, Figure 1). A single sampling point in the centre of the pond made a total of 31 sampling points at the study site (Figure 1). In the peatland area, the floating mats of *Sphagnum* were cut and removed to access the underlying water. Around 50 mL of water were collected at each sampling point by submerging sterile 50 mL Falcon tubes in the water (sampling depth: 10 cm below water surface). The Falcon tubes were then stored at 4 °C in the dark until filtration within two hours. Littoral SW, NW and N thermokarst pond samples (4 m) were cloudy pink in colour. Between 10 and 30 mL of water were filtered through 0.22 µm pore-size Sterivex^TM^ filter units (until clogging of the filters). The filters were stored at −60 °C until nucleic acid extractions from the filters. The nucleic acids (DNA) were extracted from the Sterivex^TM^ filters (EMD Millipore, Burlington, MA, USA) using a Qiagen Allprep DNA/RNA Mini Kit (Qiagen, Hilden, Germany) with modifications [35], and then stored at −20 °C until library preparation.

### 2.2. Quantitative Polymerase Chain Reaction (qPCR) Analysis

The abundance of 16S rRNA genes from Bacteria and Archaea in the thermokarst pond and in its surrounding peatland area was estimated using quantitative PCR (qPCR) with the primers Bact1369f/Bact1492r [36] and Arc787f/Arc1059r [37], respectively. The abundance of aerobic methanotrophs and methanogens was also estimated by quantification of the *pmoA* gene using the pmoA-189-f/pmoA-661mb-r primer set [38] and the *mcrA* gene using the mlas-mod-f/mcrA-rev-r primer set [39,40], respectively. Quantifications were performed in triplicate with different template concentrations (0.5, 1, 1.5 ng of DNA) to compensate for potential PCR inhibition. Amplification reactions were carried out in a 7500 Fast Real-Time system (Applied Biosystems, Foster City, CA, USA) in a final volume of 25 μL using Brilliant III Ultra-Fast Q-PCR Master Mix (Agilent, Santa Clara, CA, USA), optimal concentration of primers (0.5 μM for archaeal 16S rRNA gene, 0.6 μM for bacterial 16S rRNA gene and 1.1 μM for *pmoA* and *mcrA* genes) and 5 μL of DNA template. For 16S rRNA quantification, qPCR conditions were as follows: 40 cycles of denaturation at 95 °C for 15 s then annealing and extension at 60 °C for 60 s. For *mcrA* and *pmoA* gene quantification, annealing was carried out at 55 °C for 30 s then fluorescence was measured at 72 °C for 30 s during each cycle. Standard curves were prepared in triplicate with dilutions ranging from 0.001 to 100 nM of DNA extracted from *Methylomonas methanica* (DSM25384) for bacteria and methanotroph quantifications and *Methanosarcina acetivorans* (DSM2834) for archaea and methanogen quantifications. The R^2^ of standard curves obtained by qPCR were above 0.99 and PCR efficiencies above 90.5%. The qPCR results were expressed in terms of number of genes per milliliter of sampled water.

### 2.3. Illumina MiSeq Amplicon Library Preparation, Sequencing and Analysis

The microbial community composition of the water samples was determined by high throughput MiSeq Illumina sequencing of bacterial and archaeal 16S rRNA genes using V4 primers for Bacteria S-D-Bact-0516-a-S-18/S-D-Bact-0907-a-A-20 [41] and V1–V3 primers for Archaea A27F/Arc518R [42]. All PCR reactions were conducted in triplicate with negative controls using Brilliant III Ultra-Fast Q-PCR Master Mix, 0.5 μM of each primer and 1 μL of DNA template in a 25 μL reaction. The PCR assay comprised 30 cycles of denaturation at 95 °C for 15 s, annealing at 58 °C for 30 s and extension for 30 s at 72 °C followed by a final extension step at 72 °C for 7 min. Replicate amplicons were pooled and PCR products were indexed during a second PCR as previously described [43]. Amplicons were purified on agarose then sequenced using an Illumina MiSeq v3 kit at the IBIS/Université Laval, Plate-forme d’Analyses Génomiques (Québec, QC, Canada). Reads were assembled into single pair-end sequences and curated as detailed in a GitHub repository (https://github.com/CruaudPe/MiSeq_Multigenique). Overlapping paired-end reads were reassembled using FLASH v.2.2.00 [44] with default settings and extended maximum overlap length (300). CUTADAPT v.2.12 [45] with default settings was used to remove primers. Sorted sequences were dereplicated, clustered into operational taxonomic units (OTUs, 97% similarity) and putative chimeric sequences and singletons were removed using VSEARCH v.2.3.4 [46]. Taxonomic affiliations of the reads were determined with Mothur [47] using BASTn against Silva database release 128 [48]. OTUs belonging to lineages with cultivated or genomic representatives involved in methanogenesis and methanotrophy were isolated and analysed in detail. Raw sequences were deposited in the NCBI public database under Bioproject PRJNA527308.

### 2.4. Statistical Analyses

Statistical analyses of the data set (Mann–Whitney test, non-parametric multivariate analysis of variance (NP-MANOVA), similarity percentages breakdown procedure (SIMPER), and Bray–Curtis–based non-metric multidimensional scaling (NMDS)) were carried out according to recommendations of the Guide to Statistical Analysis in Microbial Ecology [49], using PAST software [50]. For statistical and diversity tests (NMDS, NP-MANOVA, SIMPER, OTU distribution), the results from amplicon sequencing were first normalized by rarefaction to the sample with the fewest reads, resulting in 14,241 bacterial and 4601 archaeal reads per sample.

## 3. Results

### 3.1. Microbial Abundance

**Figure 2:**
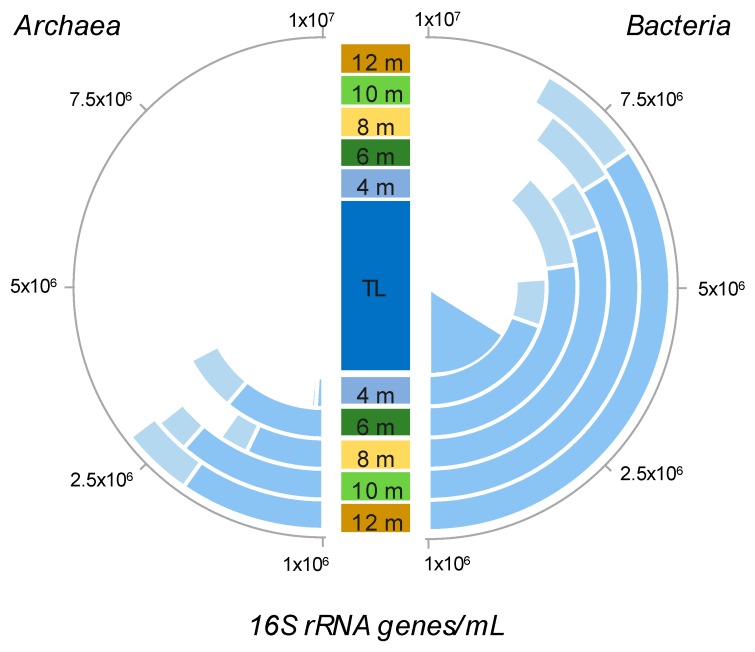
Relative abundance of *Archaea* and *Bacteria* based on 16S rRNA gene quantification. Blue bars represent the average values (*n* = 6) and light blue bars the maximum values. TL: centre of the thermokarst pond.

The qPCR results showed that *Archaea* + *Bacteria* (total prokaryote) 16S rRNA gene abundance increased from the centre of the pond outwards, from 1.95 × 10^6^ 16S rRNA genes mL^−1^ in the centre to an average of 7.05 × 10^6^ 16S rRNA genes mL^−1^ 12 m from the centre (Figure 2). In the thermokarst pond (centre and littoral (4 m) sampling points), bacterial 16S rRNA genes represented more than 96% of the total 16S rRNA gene abundance with an average of 2.3 × 10^6^ 16S rRNA genes mL^−1^, and around 82% of the total 16S rRNA gene abundance in the peatland water with an average of 5.16 × 10^6^ 16S rRNA genes mL^−1^. Archaeal 16S rRNA gene numbers were low in the thermokarst pond with less than 1 × 10^5^ 16S rRNA genes mL^−1^, but increased in the peatland waters with an average of 2 × 10^6^ 16S rRNA genes mL^−1^ (Figure 2).

### 3.2. Bacterial and Archaeal OTU Distribution

**Figure 3:**
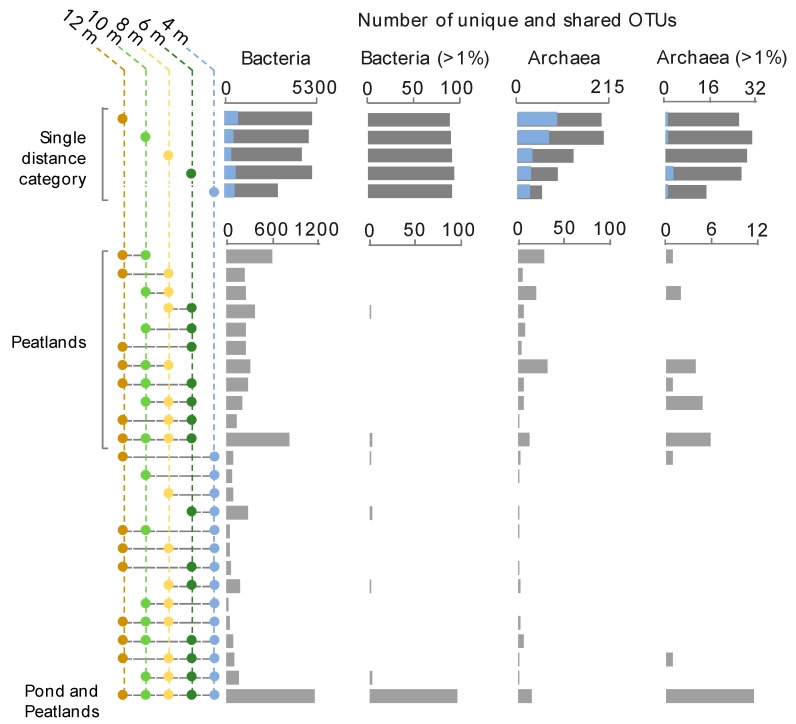
Number of OTUs detected across the sampling site. The upper graphs show values for single distance categories (specific distance locations); the grey bars represent the total number of different OTUs and predominant OTUs (abundance > 1%) detected in that category. The blue bars represent the number of OTUs that were unique to that specific distance category. The lower graphs show the number of OTUs that were shared among combinations of single areas. The centre of the thermokarst pond was excluded so that all distance categories had the same number of samples.

The distribution of bacterial and archaeal OTUs along the gradient from the centre to 12 m away was estimated as the sum of OTUs (defined at 97% sequence similarity) in samples from each distance category (4, 6, 8, 10, or 12 m) (“Single distance category” in Figure 3). For *Bacteria*, 4710 OTUs (97% sequence similarity) and 100 predominant OTUs (defined as OTUs representing more than 1% of the reads in at least one sample) were detected on average. Up to 1200 OTUs and almost all predominant OTUs (91% of the predominant OTUs) were shared among all samples (“pond and peatlands” in Figure 3). Up to 3860 OTUs were also shared between at least two peatland distance categories (“Peatland areas” in Figure 3). For *Archaea*, the number of OTUs detected per category increased with distance from the pond, with 60 OTUs in the pond and up to 210 OTUs 10 and 12 m away from the centre of the pond (“Single distance category” in Figure 3). A high proportion of the archaeal OTUs (40%) were only detected in a single distance category. Less than 32 predominant archaeal OTUs (representing more than 1% of the reads) were identified, most of them shared among all distance categories or only among peatland samples. For both *Bacteria* and *Archaea*, the number of shared OTUs between samples increased when distance categories were continuous (“Peatlands” and “Pond and Peatlands” in Figure 3).

### 3.3. Bacterial and Methanotroph Community Composition

**Figure 4:**
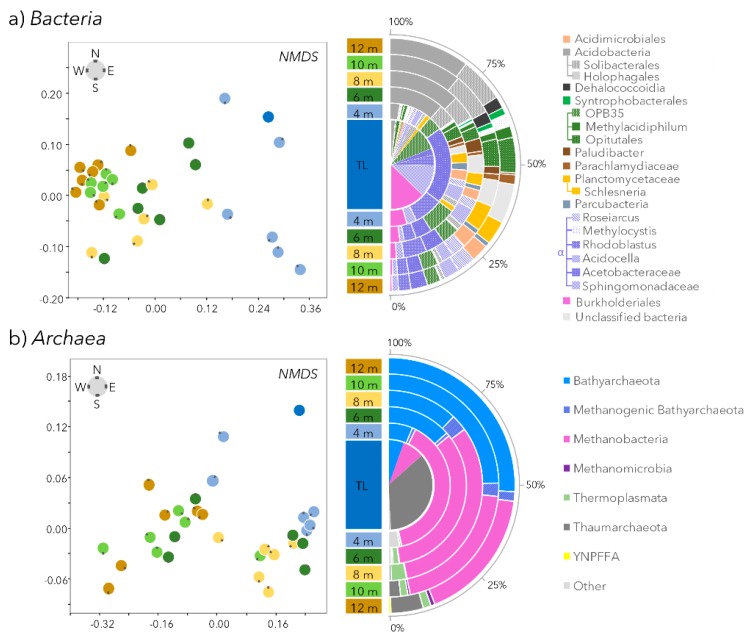
Non-metric dimensional scaling of the microbial 16S rRNA genes and the relative proportions of the major taxonomic groups shown in the half circle. (**a**) Bacterial 16S rRNA genes and their taxonomic affiliations (average of the six samples from the same distance). Colors indicate lineages: greens, *Verrucomicrobia*; yellows, *Planctomycetes*; purples, *Alphaproteobacteria*; and greys, *Acidobacteria*. (**b**) Archaeal 16S rRNA genes and their taxonomic affiliations. The color of the dots in the NMDS plot indicates the sample distance from the centre of the lake. The small black dot on each NMDS point indicates the transect of origin of the sample (N, NE, S, SE, SW, NW) and is placed according to the orientation of the compass in the top left corner. TL: centre of the thermokarst pond.

**Figure 5:**
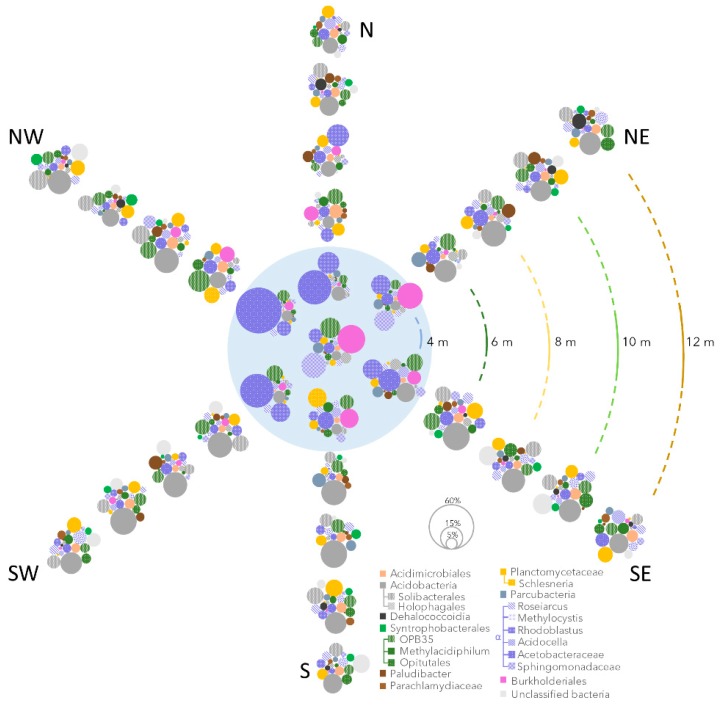
Relative proportions of bacterial 16S rRNA genes and their taxonomic affiliations, in all samples from the centre of the pond, represented as the pale blue circle, to 12 m distance across the peatland. The size of the dots represents the relative proportion of the lineages, and colors indicate the lineages: green, *Verrucomicrobia*; yellow, *Planctomycetes*; purple, *Alphaproteobacteria*; and grey, *Acidobacteria*.

Based on 16S rRNA gene sequencing, bacterial community composition changed progressively from the centre outwards (Figure 4a and Figure 5). The community from the pond (centre and the 4 m littoral samples) and peatland samples (6, 8, 10, and 12 m samples) were significantly different (NP-MANOVA, *p*-value: 0.0001). In the centre of the pond, the bacterial community was heterogeneous and dominated (mean ± SD percent total reads) by members of the *Burkholderiales* (11 ± 7% of the 16S rRNA genes), *Alphaproteobacteria* (*Sphingomonadales* (9 ± 7%), *Acetobacteraceae* (25 ± 20%), *Rhodoblastus* (7 ± 3%) and *Verrucomicrobia* OPB35 (7 ± 3%). Together, these lineages explained 26.3% of the dissimilarity between pond and peatland microbial communities (SIMPER analysis). By contrast, bacterial communities in the peatland samples (6, 8, 10, and 12 m away) were similar, and predominated by members of the *Acidobacteria* (*Acidobacteriales* (14 ± 5%), *Solibacterales* (6 ± 3%), *Acidimicrobiales* (3 ± 1%), *Acidocella* (3 ± 1%), *Roseiarcus* (2 ± 0.8%), *Planctomycetaceae* (4 ± 2%) and *Verrucomicrobia* (*Opitutales* (4 ± 1%) and *Methylacidiphilum* (1.8 ± 0.8%)) (Figure 4a and Figure 5). These lineages explained together another 25.8% of the dissimilarity between pond and peatland microbial communities (SIMPER analysis).

**Figure 6:**
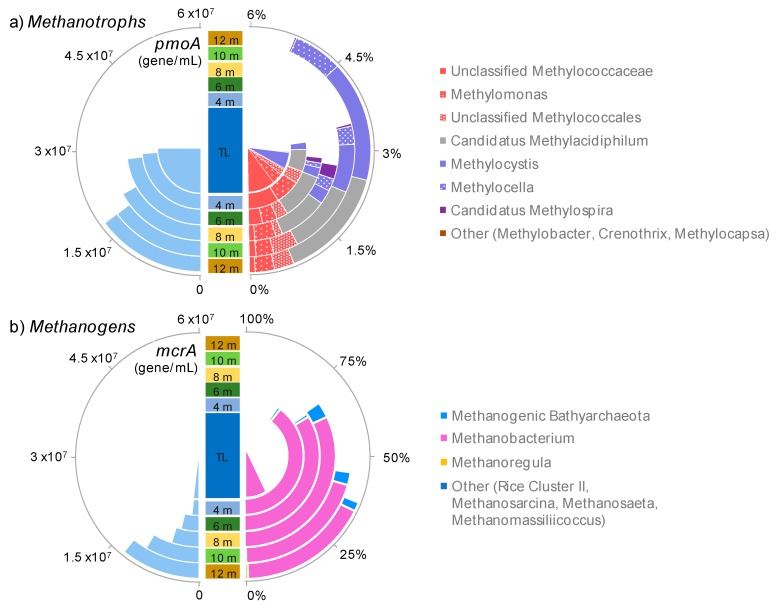
Distribution and taxonomic affinity of methane cycling genes. (**a**) Average number of *pmoA* genes per area and relative proportion and taxonomic affiliation of potential methanotrophs. (**b**) Average number of *mcrA* genes per area and relative proportion and taxonomic affiliation of potential methanogens.TL: centre of the thermokarst pond.

16S rRNA genes affiliated with potential aerobic methanotrophs represented on average 3.4% of the reads in all samples and a maximum of 5% of the bacterial 16S rRNA genes 12 m away from the centre of the pond (Figure 6a). The qPCR targeting the *pmoA* gene indicated that the abundance of potential aerobic methanotrophs was similar in the thermokarst pond and its surrounding peatland area with an average of 2.5 ± 1.6 × 10^7^ *pmoA* genes mL^−1^ (Mann–Whitney test: *p*-value: 0.13) (Figure 6a). However, 16S rRNA gene sequencing indicated that methanotrophic community changed with the distance from the pond edge (Figure 6a). The methanotroph community in the thermokarst pond was dominated by members of the *Methylococcales*, including *Methylomonas* species (2.6 ± 1.2% of the bacterial community). *Methylomonas* reads were mainly represented by an OTU that was highly similar to *Methylomonas paludis* (AJ563927; 100% sequence similarity over the 426 nucleotides), isolated from an acidic wetland [51]. By contrast, members of the *Candidatus Methylacidiphilum* (1.8 ± 0.8%), *Methylocystis* (2 ± 0.9%), and *Methylocella* (0.8 ± 0.3%) were the major methanotrophic lineages in the peatland samples (Figure 6a). *Methylocystis* and *Methylocella* OTUs were highly similar (100% sequence similarity) to sequences from peatland ecosystems [52], whereas *Ca. Methylacidiphilum* OTUs were more similar to sequences from Arctic soils [53]. Other potential methanotrophic lineages such as *Methylobacter*, *Crenothrix*, and *Methylocapsa* were also detected but in low proportions (<0.05% of the bacterial community in all samples).

### 3.4. Archaeal and Methanogen Community Composition

Archaeal community composition, determined by 16S rRNA gene sequencing, changed progressively from the centre outwards (Figure 4b). Members of *Thaumarchaeota* represented 71% of the reads in the centre of the pond but were a minority of the reads in all other samples, including littoral samples (4 m). *Methanobacteria* were the predominant lineage in the littoral water of the pond (4 m) with on average (±SD) 79 ± 24% of the reads. The relative proportion of *Methanobacteria* decreased with increasing distance from the thermokarst pond edge, to an average of 34 ± 17% of the archaeal 16S rRNA genes at the furthest ring sampled, 12 m from the centre. In contrast, the relative proportion of 16S rRNA genes related to *Bathyarchaeota* followed the opposite trend, accounting for 11 ± 8% of the total 16S rRNA genes in the pond and up to 52 ± 9% at the farthest sampling area (Figure 4b). Finally, members of the *Thermoplasmatales* were also detected in the peatland water samples but in lower relative proportions (<5% of the reads).

Several potential methanogenic lineages were detected in the thermokarst pond and its surrounding peatland (Figure 6b). The *Methanobacterium* lineage represented the predominant potential methanogens in all samples and was mainly represented by a single OTU, found in all samples. This OTU was highly similar (100% sequence identity of 441 nucleotides) to a hydrogenotrophic, moderately acidophilic *Methanobacterium* enriched from an acidic peat bog (AJ459882; [54]). In addition to *Methanobacterium*, a small proportion of *Bathyarchaeota* (<5% in all samples) were related to the putative methanogenic lineage of the *Bathyarchaeota* [55]. *Methanomicrobiales* (*Methanoregula)* and *Methanosarcinales* members (*Methanosarcina* and *Methanosaeta*) were also detected (<2%) as well as methanogenic members of the *Thermoplasmatales* (*Methanomassiliicoccus*, <2%). No anaerobic methanotrophs (ANME) 16S rRNA gene were detected. In total, the potential methanogenic lineages represented 16% of the archaeal 16S rRNA genes in the centre of the pond, 81 ± 24% of the reads in the littoral water (4m from the centre) and then decreased with distance away from the pond to 37 ± 17% of the reads at 12 m (Figure 6b). However, this decrease in relative abundance may have been compensated by the increasing overall archaeal 16S rRNA gene abundance, which was shown by qPCR (Figure 2). The qPCR analyses targeting the *mcrA* gene indicated that potential methanogen abundance increased by an order of magnitude with distance from the pond centre from, 2.1 ± 1.3 × 10^6^ *mcrA* genes mL^−1^ in the pond to 2.0 ± 0.6 × 10^7^ *mcrA* genes mL^−1^ at 12 m distance in the peatland (Mann–Whitney test; *p:* 0.01; Figure 6b). Furthermore, this quantification would have excluded methanogenic *Bathyarchaeota*, with divergent *mcrA* genes, which may not have been amplified using our standard primer set [55]. The absolute abundance of methanogens might have been underestimated in the peatland area where potential methanogenic *Bathyarchaeota* were detected by 16S rRNA gene sequencing. 

## 4. Discussion

Permafrost thawing in subarctic peatlands tends to follow a successional sequence beginning with an increase in number and area of thermokarst ponds, followed by aquatic plant colonization and peat accumulation [2,14,30]. Both ponds and peatlands are associated with greenhouse gas emissions, especially methane, at northern high latitudes [9,18,56] and for ponds the net flux is influenced by interaction of methanogens and methanotrophs in the overlying water [27]. Here we found changes in microbial communities, including taxa associated with methane cycling, along a thermokarst pond-peatland transition, suggesting that local conditions select for specific archaeal and bacterial communities, which would in turn potentially influence net methane emissions.

### 4.1. Microbial Continuum across the Wetland Ecosystem

Our analyses indicated that a high proportion of bacterial and archaeal OTUs were shared in the pond and peatland waters, at least as far as 12 m from the centre of the pond (Figure 3), indicating microbial connectivity and a continuum from the pond into the surrounding wetland. This microbial continuity suggests ongoing exchanges between the pond and the surrounding wetland. The progressive change of community structure highlighted by NMDS (Figure 4 and Figure 5), along with the higher number of shared OTUs between adjacent distance categories (Figure 3), were consistent with such connectivity.

Superimposed on the overall tendency of similarity among distance categories, we detected spatial variability in microbial community composition within the pond, especially in the littoral samples (4 m, blue dots in Figure 1 and NMDS, Figure 5), which suggests divergent environmental conditions within this zone. Unfortunately, due to logistic constraints, detailed physical and chemical data from this pond on the day of sampling is not available. However, since the community variability was mainly due to the overrepresentation in SW, NW, and N littoral samples (>34% of the bacterial 16S rRNA genes) of a single OTU affiliated with the *Acetobacteraceae*, which had 97% sequence similarity with *Acidisphaera rubrifaciens*, an aerobic, acidophilic, chemo-organotrophic bacteria with bacteriochlorophyll, and potentially involved in plant-derived organic polymer degradation [57], this result suggests a strong influence of the aquatic vegetation on thermokarst pond microbial community composition near the shore. More broadly, the variability near the shore within a single thermokarst pond implies that the community composition extrapolated from a single sample may not capture the entire pond biodiversity and there is a need for spatial replicates in future studies comparing ponds and regions. 

### 4.2. Microbial Community Change along the Thermokarst Pond–Peatland Continuum

Microbial community composition changed with the distance from the pond centre, regardless of the transect direction, supporting the inferred influence of the aquatic vegetation on microbial community composition and potential functions (Figure 4 and Figure 5). For instance, the colonization of the open water by aquatic plants would limit light availability for phototrophic bacteria such as the bacteriochlorophyll-producing *Burkholderiales*, and *Alphaproteobacteria* (*Sphingomonadales*, *Acetobacteraceae* and *Rhodoblastus*) [58]), and thereby affect the community structure. Beta-diversity analysis (NMDS using Bray–Curtis indices, Figure 4) highlighted a stronger similarity between microbial communities of the peatland samples from the same area (6, 8, 10, or 12 m) compared to the thermokarst pond samples (Figure 5). This result suggests that the peatland ecosystem strongly selected the microbial communities. In addition, the reduced variability in the wetland communities surrounding the pond suggests that peatlands were exposed to less environmental fluctuation than in the open water (Figure 4 and Figure 5). The higher microbial abundance (*t*-test; *p*-value < 0.001) in the peatlands would also indicate lower loss rates compared to the pond environment, where heterotrophic microbial eukaryotes (including predators) would be abundant.

Consistent with a steadier environment with a greater variety of micro-environmental niches, the peatland waters had significantly higher numbers of OTUs (*t*-test; *p*-value: <0.001) compared to the thermokarst pond (Figure 2, Figure 3, Figure 4 and Figure 5). The relative proportion of *Acidobacteria*, and other lineages with acidophilic proprieties (e.g., *Acidimicrobiales*, *Acidocella, Methylocystis*, *Ca.* Methylacidiphilum) also increased with distance from the centre (Figure 4 and Figure 5). Acidophilic bacteria have been frequently reported in peatlands [59,60] and are likely to be selected by the acidification of the water by *Sphagnum* mosses that contain high concentrations of organic acids such as sphagnol and uronic acid polymers in their cell walls [61] and that exchange protons for other cations in the water. Furthermore, the micro-topographic gradient could also contribute to the acidification of the most distant samples [34] and the enrichment of acidophilic microorganisms revealed by 16S rRNA gene sequencing (Figure 4 and Figure 5). Therefore, the accumulation of organic matter and the acidification of the water by humified vegetation including *Sphagnum* would likely be the main drivers of the changes in microbial community composition and function associated with the peatland progression over the thermokarst pond. The relative abundance of methanogens as well as relative proportion of bacterial lineages with anaerobic metabolism and capacity for organic carbon degradation (*Opitutales* [62]) and syntrophy with methanogens (*Syntrophobacteraceae*, *Dehalococcoidia*, [63]) also increased with distance in the peatland area (Figure 4, Figure 5 and Figure 6), suggesting the increasing occurrence of anaerobic and methanogenic niches in the peatland waters, as previously reported in other peatlands [59].

### 4.3. Methane Cycling in the Changing Landscape

Quantification of the *mcrA* and *pmoA* genes combined with 16S rRNA gene sequencing showed the presence of methanogens and aerobic methanotrophs in all water samples (Figure 4, Figure 5 and Figure 6), indicating that methane cycling occurs in both the thermokarst and peatland environments. The methanogenic community composition changed little and was similar between the surface water of the thermokarst pond and peatland area, with a single predominant OTU affiliated with a moderate acidophilic, H_2_:CO_2_-dependent *Methanobacterium* [54]. Despite their sensitivity to oxygen, methanogens are usually detected from the surface waters of the thermokarst lake down to the sediments [9,10,64,65]. Our results, based on surface waters, might not reflect the entire methanogenic community of the thermokarst pond. Nonetheless, the predominant methanogen in this study differs from methanogens identified in surface and bottom waters of neighbouring, but deeper, larger and less acidic (pH > 6) lakes that were related to *Methanomicrobiales* and *Methanosarcinales* [10,33,64]. In contrast, the taxon *Methanobacterium* is frequently identified as the main lineage of methanogens in acidic peatlands [66,67,68,69]. This result, associated with the increase of methanogen abundance in the peatland area observed by quantitative PCR, suggests a modification of the methane producing community by the acidic peatland environment and the aquatic plant colonization, toward acidophilic and hydrogen-consuming lineages (Figure 6a,b). 

In contrast to the methanogens, methanotroph abundance did not change with distance from the centre of the pond (Figure 6), however their community composition shifted over the transition into the peatland waters (Figure 4 and Figure 5). Members of *Methylococcales* were the predominant methanotrophs in the thermokarst pond, as previously reported [23,27], but their relative proportion decreased under the floating vegetation, as observed in European boreal peatlands [28]. By contrast, potential acidophilic lineages of methanotrophs, including *Ca*. Methylacidiphilum and *Methylocystis*, were rare in the pond but predominated in the peatland water community (Figure 4 and Figure 5), supporting previous observations in other peatlands [28,52,70] and the influence of the peatland environmental conditions on methane cycling microorganisms observed with the methanogenic community. Detection of methanotrophic species related to *Methanocystis* in the peatland water is also consistent with their frequent occurrence in dead cells of *Sphagnum* mosses, where conditions are optimal for pH, oxygen, and methane [29].

Although the DNA-based abundance and diversity of methane cycling prokaryotes may not translate directly into methane emission flux rates, our results indicate that methanogenic and methanotrophic communities were affected differently by the distance from the edge of the pond and closely tracked the peatland plant succession over the thermokarst waterbody. This underscores the need for improved spatial resolution and monitoring of peatlands to accurately assess the net greenhouse gas emissions from the coupled thermokarst pond-peatland biome of the vast northern landscape.
